# Lichen planus pemphigoides treated with a low dose of oral prednisolone and omalizumab

**DOI:** 10.1016/j.jdcr.2024.08.039

**Published:** 2024-09-19

**Authors:** Yurie Matsuura, Yuki Mizutani, Makoto Kondo, Keiichi Yamanaka

**Affiliations:** Department of Dermatology, Graduate School of Medicine, Mie University, Tsu, Japan

**Keywords:** BP180, BP230, IgE, IgE receptor, Lichen planus pemphigoides, omalizumab, palmoplantar keratoderma

## Introduction

Autoimmune blistering diseases can exhibit a range of clinical symptoms, necessitating precise diagnosis and tailored treatment. Bullous pemphigoid (BP) and lichen planus pemphigoides (LPP) are such diseases, characterized by overlapping pathological features yet distinct clinical trajectories and responses to treatment.

In this case report, BP was initially suspected based on the initial clinical presentation. However, the presence of atypical symptoms and pathological features ultimately led to a diagnosis of LPP. Given the involvement of immunoglobulin E (IgE) antibodies, treatment was initiated with omalizumab. This report suggests that omalizumab may serve as an effective treatment option for certain autoimmune blistering diseases.

## Case report

**Fig 1:**
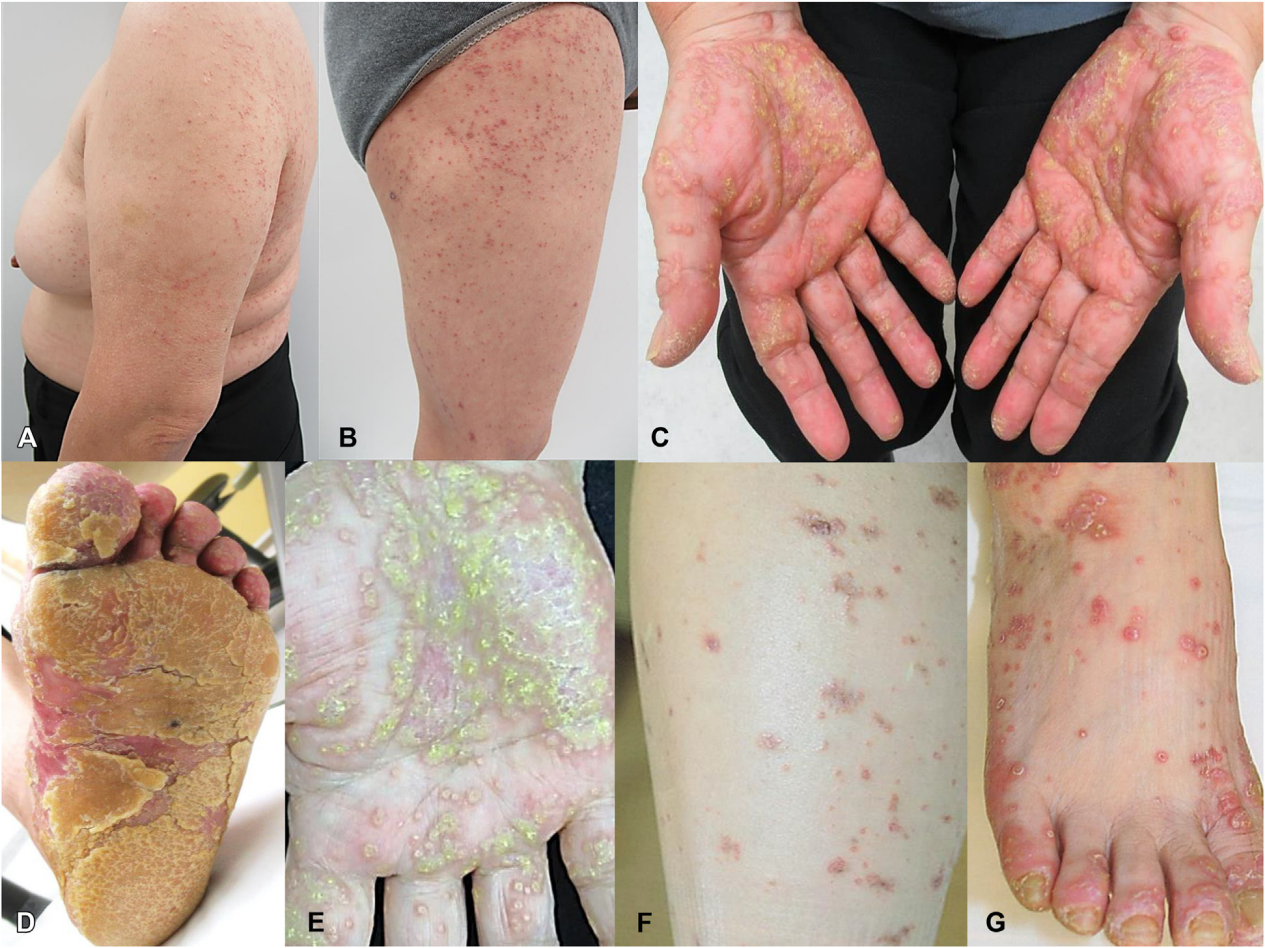
Clinical presentation at the first visit to our hospital (**A-F**). Pruritic papular eruptions with small pustules on limbs and trunk (**A** and **B**); hyperkeratosis on palms and soles (**C-E**); papular and nodular eruptions and blisters on the legs and dorsum of the foot (**F** and **G**).

**Fig 2:**
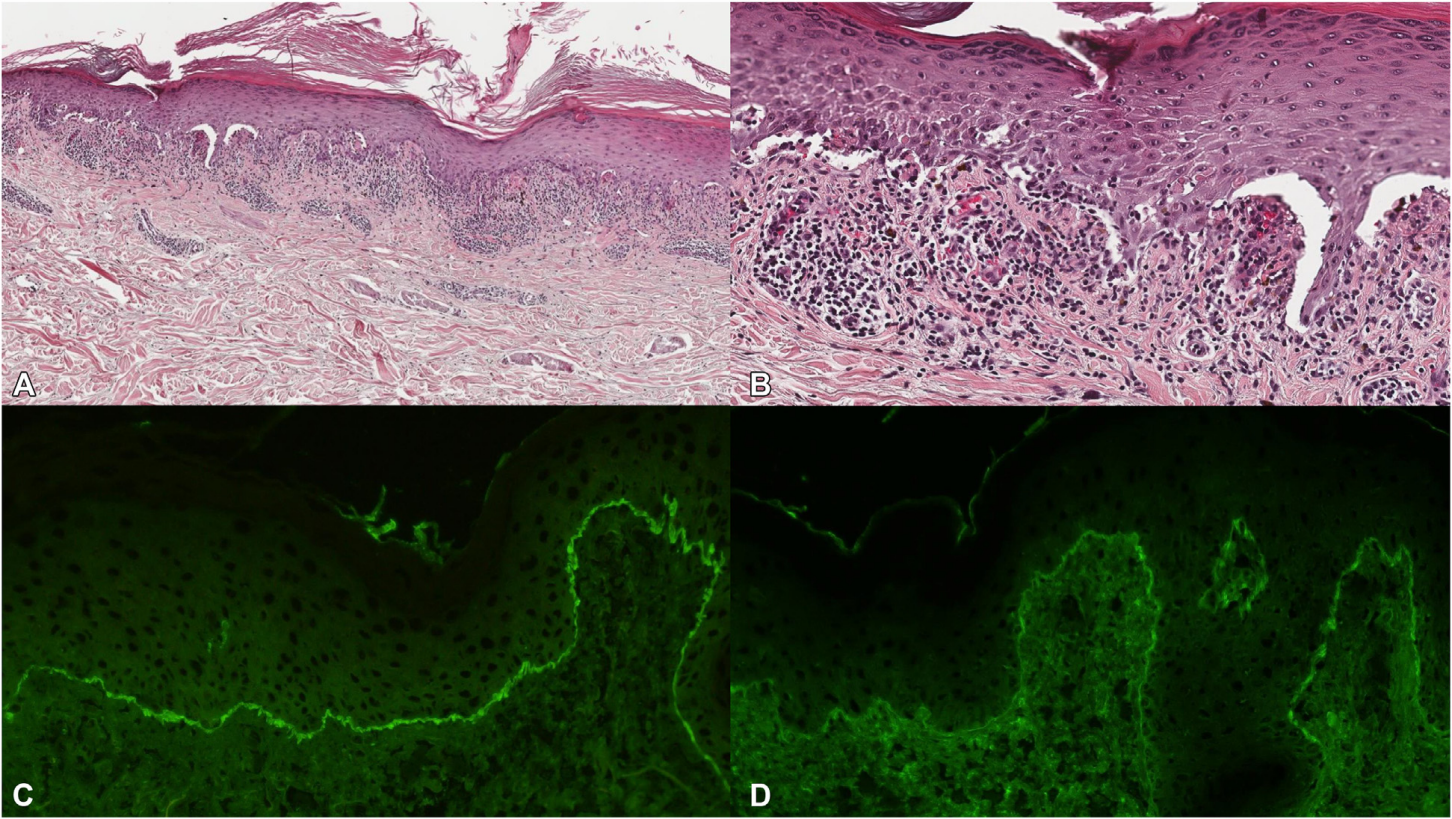
Histopathological findings showed saw-tooth epidermal hyperplasia, hyperkeratosis, subepidermal cleft formation, and lymphocytic infiltration at the dermoepidermal junction (hematoxylin-eosin, original magnification **A** ×40, **B** ×200). DIF showed linear IgG (**C**) and C3 (**D**) deposits at the basal membrane zone. *DIF*, Direct immunofluorescence; *IgG*, immunoglobulin G.

**Fig 3:**
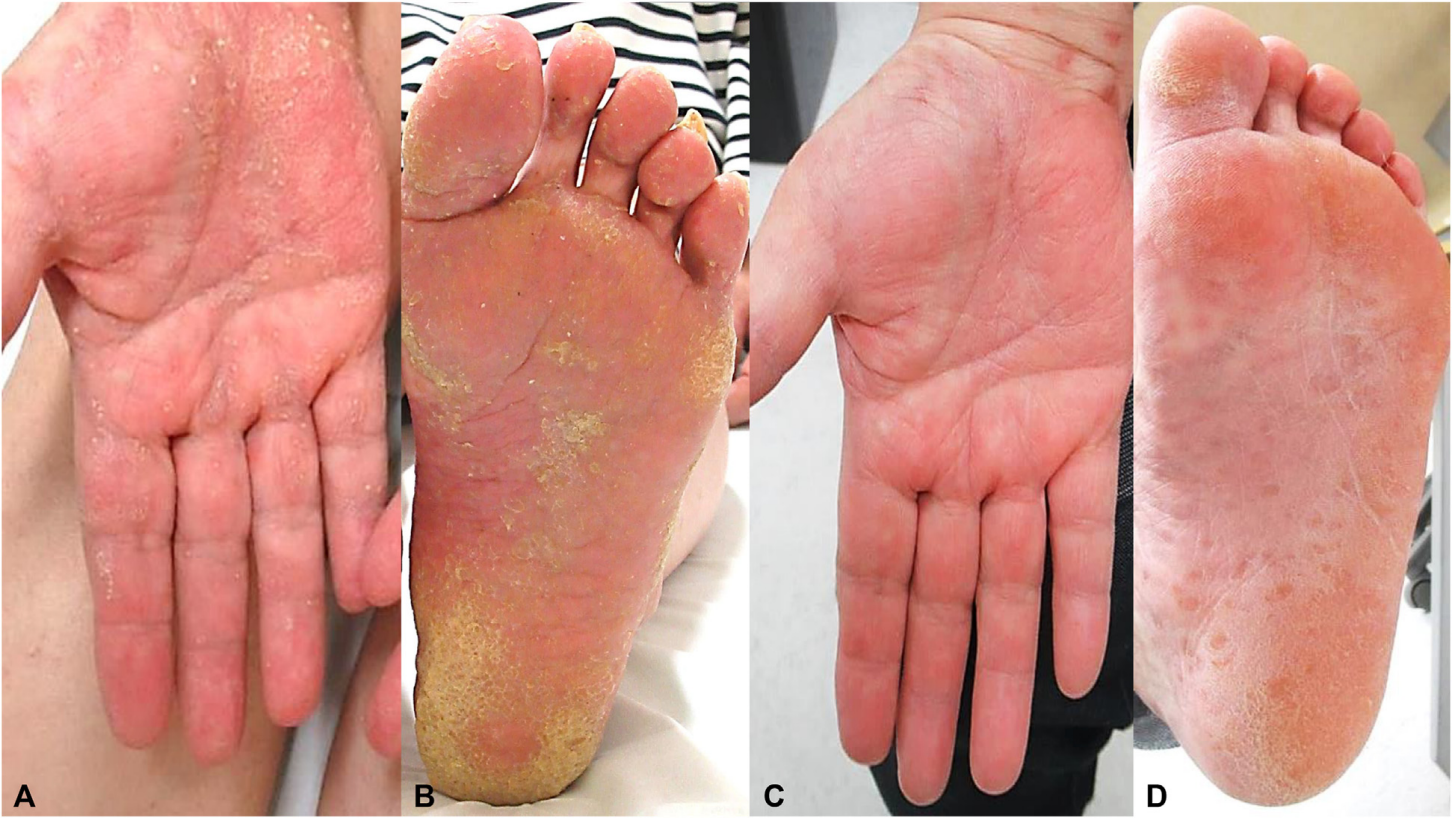
Clinical presentation after the initiation of prednisolone at 20 mg/day and omalizumab treatment. All skin lesions had diminished 2 weeks after the initiation of treatment (**A** and **B**) and almost disappeared after another 12 weeks (**C** and **D**).

A 63-year-old female initially presented with erythema and blisters on her toes 6 months prior to her initial consultation at our institution. The dermatological manifestations progressively extended to her hands and feet, accompanied by palmoplantar hyperkeratosis. Furthermore, pruritic nodular eruptions with small pustules developed on the limbs, followed by the spread of lichenoid papules on the trunk. (Fig 1). Two skin biopsies were conducted: one from a blister located on the leg and another from a nodular eruption on the thigh. Histopathological findings revealed saw-tooth epidermal hyperplasia and hyperkeratosis, with subepidermal cleft formation and lymphocytic infiltration along the dermoepidermal junction (Fig 2, *A* and *B*). Direct immunofluorescence demonstrated linear immunoglobulin G (IgG) and C3 deposition along the basal membrane zone associated with a nodular eruption (Fig 2, *C* and *D*). Laboratory investigations revealed elevated eosinophil counts (270/μL), increased IgE levels (329 IU/mL), and elevated IgG anti-BP180 antibodies (49.8 U/mL). We initially considered it BP because of the presence of erythema and blisters and the positive result for anti-BP180 antibodies; however, based on the atypical clinical presentation and histopathological findings, led to the diagnosis of LPP. Initial treatment with topical steroids did not yield improvement; thus, a regimen including prednisolone at a dosage of 20 mg/day (0.3 mg/kg/day) and monthly subcutaneous administration of 300 mg of omalizumab was initiated. Two weeks after initiating this treatment regimen, a remarkable reduction in pruritus and significant diminution of all cutaneous lesions were observed (Fig 3, *A* and *B*). Furthermore, all types of skin lesions had almost completely resolved after an additional 12 weeks (Fig 3, *C* and *D*). Subsequently, it was possible to gradually taper the prednisolone dose without any recurrence of the dermatological manifestations.

## Discussion

LPP is an autoimmune blistering disease that presents clinically with lichenoid features associated with lichen planus, alongside blisters characteristic of bullous pemphigoid. There are clinical, histological, and immunological overlaps between LPP and BP, attributable to autoimmune reactions against collagen XVII (COL17)—a component of the hemidesmosomes in basal keratinocytes. This overlap is evidenced by linear deposits of IgG or C3 at the basement membrane zone observed on direct immunofluorescence. Nevertheless, subtle differences exist in the clinical presentations of BP and LPP. Notably, the typical onset age for LPP is significantly younger than that of BP. Moreover, LPP lesions primarily appear on the extremities, in contrast to the more generalized distribution and associated pruritus seen in BP cases. Although systemic corticosteroids have successfully treated in reported cases of LPP, concerns about side effects have led researchers such as Hübner et al to propose immunosuppressive therapies, including cyclosporine and methotrexate, as alternatives or adjuncts to corticosteroid treatment.^1^

In the current case, we observed the combination of palmoplantar keratoderma and pruritic papular and nodular eruptions on the limbs, which is atypical for BP, despite IgG anti-BP180 antibodies being elevated. Literature review reveals that palmoplantar hyperkeratosis has been documented to occur in association with bullous disorders.^2^ Altered cell adhesion may precipitate abnormal keratinization through enhanced keratinocyte turnover, culminating in the retention of the cornified layer. Consequently, the disruption in cell adhesion could contribute not only to the formation of blisters but also to aberrant keratinization processes.

Historically, the pathogenesis of BP, the most common autoimmune blistering disease, was primarily attributed to the presence of IgG autoantibodies targeting basal membrane proteins, specifically BP180 and BP230. Recent studies, however, have underscored the significance of specific IgE antibodies against the same proteins, BP180 and BP230, in the development of BP.^3^ Ujiie et al have noted that BP180 or BP230-specific IgE antibodies, independent of IgG-mediated autoimmunity, are demonstrated to bind at the epidermal-dermal junction, inducing skin erythema and eosinophil infiltration via eosinophil degranulation in lesional tissue.^4^ Based on these considerations, numerous successful treatments of BP using omalizumab, a humanized monoclonal antibody that prevents IgE from binding to its high-affinity receptor (FcεR1), have been reported.^5^, ^6^, ^7^ The efficacy of omalizumab in specific instances of BP may be attributed to the expression of high-affinity IgE receptors on human eosinophils.^5^ Omalizumab is included as a Grade 4 recommendation in the guidelines for treatment-resistant BP patients in Europe, Italy, and Germany. Additionally, it was cited in the recently updated 2021 French guidelines.^7^ Fania et al observed elevated serum IgE levels in patients with BP manifesting symptoms akin to palmoplantar keratoderma. Additionally, it was noted that such patients exhibited resistance to steroid therapy.^1^ In the present case, the observation of primarily pruritic erythema with minimal blister formation along with confirmed elevated IgE levels led us to consider the role of IgE antibodies. Consequently, increased total IgE levels and peripheral eosinophil counts decreased, and skin lesions improved after initiating omalizumab with steroids. Moreover, despite administering a low dose of prednisolone, a prompt and significant improvement in symptoms was observed.

High doses of steroids carry risks of various complications, and there is concern about the onset of delirium during long-term hospitalization. Given this context, the use of omalizumab is anticipated to be a viable option not only for steroid-resistant autoimmune blistering disease patients but also for elderly patients who wish to avoid high doses of steroids. This report not only presents a rare case of LPP but also suggests that omalizumab may enhance the effectiveness of traditional steroid therapy in treating autoimmune blistering diseases such as LPP and BP.

## Conflicts of interest

None disclosed.
